# *ARID1A* genomic alterations driving microsatellite instability through somatic* MLH1* methylation with response to immunotherapy in metastatic lung adenocarcinoma: a case report

**DOI:** 10.1186/s13256-020-02589-1

**Published:** 2021-02-19

**Authors:** Mercedes Durán, Iris Faull, Enrique Lastra, Jean-Francois Laes, Ana Belén Rodrigo, Ricardo Sánchez-Escribano

**Affiliations:** 1 Instituto de Biología Y Genética Molecular, IBGM University of Valladolid, Sanz Y Fores Street, 3, 47003 Valladolid, Spain; 2 Guardant Health, 505 Penobscot Dr, Redwood, CA 94063 USA; 3 Molecular Tumor Board, Genetic Counselling Unit, Medical Oncology Department, Hospital Universitario de Burgos, Av. Islas Baleares, 3, 09006 Burgos, Spain; 4 ONCODNA, Rue Louis Breguet 1, 6041 Gosselies, Belgium; 5 Medical Oncology Department, Hospital Clínico Universitario De Valladolid, Av. Ramón Y Cajal, 3, 47003 Valladolid, Spain

**Keywords:** Microsatellite instability, *MLH1*, Lung adenocarcinoma, Immunotherapy, Liquid biopsy, Molecular screening, *ARID1A* gene, NGS platforms

## Abstract

**Background:**

Tumor molecular screening allows categorization of molecular alterations to select the best therapeutic strategy. AT-rich interactive domain-containing 1A (*ARID1A*) gene mutations are present in gastric, endometrial, and clear cell ovarian tumors. Inactivation of this gene impairs mismatch repair (MMR) machinery leading to an increased mutation burden that correlates with microsatellite instability (MSI), associated with tumor-infiltrating lymphocytes and programmed death ligand 1 (PD-L1) expression. This is the first case report in lung adenocarcinoma of *ARID1A* gene alterations leading to sporadic MSI, through somatic mutL homolog 1 (*MLH1*) promoter methylation, with an *MLH1* gene mutation as the second somatic hit.

**Case presentation:**

A 50-year-old never-smoker Bulgarian woman, with no comorbidities and no family history of cancer, was diagnosed with metastatic lung adenocarcinoma. PD-L1 immunohistochemistry (IHC) of tissue biopsies on right groin adenopathies resulted in 30% positivity. Liquid biopsy test reported actionable alterations in *ARID1A* gene, rearranged during transfection (*RET*) gene fusions, epidermal growth factor receptor (*EGFR*) gene R776H mutation, breast cancer (*BRCA*) genes 1/2, and cyclin-dependent kinase inhibitor 2A (*CDKN2A*) gene mutations. The patient was treated with immunotherapy, and showed a treatment response lasting for 19 months until a new metastasis appeared at the right deltoid muscle. Genomic analysis of a sample of this metastasis confirmed PD-L1 positivity of greater than 50% with CD8^+^ T cells expression and showed MSI with a deleterious c.298C>T (p.R100*) *MLH1* gene mutation. Multiplex ligation-dependent probe amplification (MLPA) of this sample unveiled *MLH1* gene promoter methylation. The *MLH1* gene mutation and the *MLH1* gene methylation were not present at the germline setting.

**Conclusions:**

In this particular case, we show that *ARID1A* gene mutations with sporadic MSI due to somatic *MLH1* gene promoter methylation and *MLH1* gene mutation could change the prognosis and define the response to immunotherapy in a patient with lung adenocarcinoma. Comprehensive solid and liquid biopsy tests are useful to find out resistance mechanisms to immune checkpoint inhibitors. Our data encourages the development of new therapies against *ARID1A* mutations and epigenomic methylation when involved in MSI neoplasms.

## Introduction

Tumor molecular screening allows categorization of molecular alterations to select the best therapeutic strategy. Targeted drugs have marked and durable efficacy in advanced lung adenocarcinoma. In the absence of these specific biomarkers, systemic immune checkpoint inhibitors are also active against neoplasms with other biological profiles: microsatellite instability (MSI), high tumor mutational burden (TMB), or programmed death ligand 1 (PD-L1) expression. Identification of molecular mechanisms for immunotherapy response can be helpful to clinicians choosing this kind of treatment. AT-rich interactive domain-containing 1A (*ARID1A*) gene mutations are known to occur in gastric, endometrial, and clear cell ovarian tumors. Inactivation of this gene impairs mismatch repair (MMR) machinery leading to an increased mutation burden that correlates with MSI, associated with tumor-infiltrating lymphocytes and PD-L1 expression. This is the first case report in lung adenocarcinoma of *ARID1A* gene alterations leading to sporadic MSI, through somatic *MLH1* promoter methylation, with an *MLH1* gene mutation as the second somatic hit, showing a clinical and radiologic response to an immune checkpoint inhibitor.

## Case description

A 50-year-old never-smoker Bulgarian woman, with no comorbidities and no family history of cancer, was diagnosed in June 2015 with stage IV lung adenocarcinoma metastatic to the peritoneum, retroperitoneum, adrenal glands, iliac and inguinal lymph nodes, as revealed by physical examination and computed tomography (CT) scan. Tissue biopsies from the primary tumor and right groin adenopathies revealed an adenocarcinoma, with positive cytokeratin-7 (CK7), epithelial membrane antigen (EMA), thyroid transcription factor-1 (TTF-1) immunohistochemistry (IHC), and negative cytokeratin-20 (CK20) staining. Real-time polymerase chain reaction (RT-PCR) (COBAS 4800 system) showed no epidermal growth factor receptor (*EGFR*) or v-RAF murine sarcoma viral oncogene homolog B (*BRAF*) gene V600E mutations. Fluorescence in situ hybridization (FISH) did not detect anaplastic lymphoma kinase (*ALK*) gene fusions, proto-onocogene 1 receptor tyrosine kinase of ROS (*ROS-1*) gene rearrangements, or tyrosine-protein kinase Met (hepatocyte growth factor receptor) (*MET*) gene amplifications. In July 2015, she started chemotherapy with cisplatin plus pemetrexed, developing adrenal insufficiency secondary to bilateral adrenal metastases, which required glucocorticoid and mineralocorticoid supplementation. Four cycles later, a partial response (PR) by Response Evaluation Criteria in Solid Tumors (RECIST) version 1.1 was obtained. After maintenance treatment with pemetrexed for 12 cycles, in July 2016 radiological progressive disease (PD) was documented at retroperitoneum and adrenal glands. In August 2016 and September 2016, three cycles of docetaxel were administered with growing metastasis only at the left adrenal gland.

With the advent of the ChekMate057 results [1], immunotherapy was proposed. PD-L-1 IHC (DAKO 22C3 antibody) on right groin adenopathies resulted in positivity of 30% and tumor sample was exhausted. To make sure the patient did not have any actionable genomic alteration, a comprehensive liquid biopsy Guardant360 test was performed. It reported 97 genomic variants with 19 actionable alterations (six at *ARID1A* gene,* RET* fusions,* EGFR* R776H mutation,* BRCA1/2* and* CDKN2A *mutations) (Table 1).

**Table 1:** Guardant360 liquid biopsy genomic alterations

*ARID1A* (%cfDNA)	*RET* (%cfDNA)	*STK11* (%cfDNA)	*NFE2L2* (%cfDNA)	*BRCA* (%cfDNA)	*EGFR* (%cfDNA)	Other genes (%cfDNA)
Exon 18 deletion (16.9%)	*KIF5B*-*RET* fusion (9.1%)	Exon 6 deletion (2.2%)	R34Q (2.1%)	Exon deletion BRCA2 (1.4%)	R776H (0.8%)	Splice site SNV NF1 (1.1%)
Exon 1 deletion (12.1%)			L30F (1.4%)	R2784Q BRCA2 (0.2%)		R181C TP53 (1.2%)
Exon 1 deletion (1.9%)				Exon 11 deletion BRCA2 (0.1%)		EXON 2 insertion CDKN2A (0.4%)
Exon 1 insertion (1.2%)				Exon 10 deletion BRCA1 (0.2%)		H83Y CDKN2A (0.2%)
R1722 (0.6%)						
Exon 20 deletion (0.4%)						

Guardant360 liquid biopsy results. 97 genomic alterations (19 actionable). 33.6% of altered cell-free DNA (%cfDNA) for five different exon deletions and one mutation in AT-rich interactive domain-containing 1A (*ARID1A*) gene. Other potential molecular targets:* RET* fusions,* BRCA1/2* gene mutations,* EGFR* R776H mutation, and* CDKN2A *mutations. 77 alterations (not depicted in the table) were VUS (variants of unknown significance), synonymous, or non-actionable mutations in different genes

*ARID1A* AT-rich interactive domain-containing 1A, *RET* ret proto-oncogene, *STK11* serine/threonine kinase 11, *NFE2L2* nuclear factor erythroid 2 like 2, *BRCA* breast cancer gene, *EGFR* epidermal growth factor receptor, *KIF5B* kinesin family member 5B, NF1 neurofibromatosis type I, *TP53* tumor protein 53, *CDKN2A* cyclin dependent kinase Inhibitor 2A, *cfDNA* cell free DNA, *SNV* single nucleotide variant

**Fig. 1:**
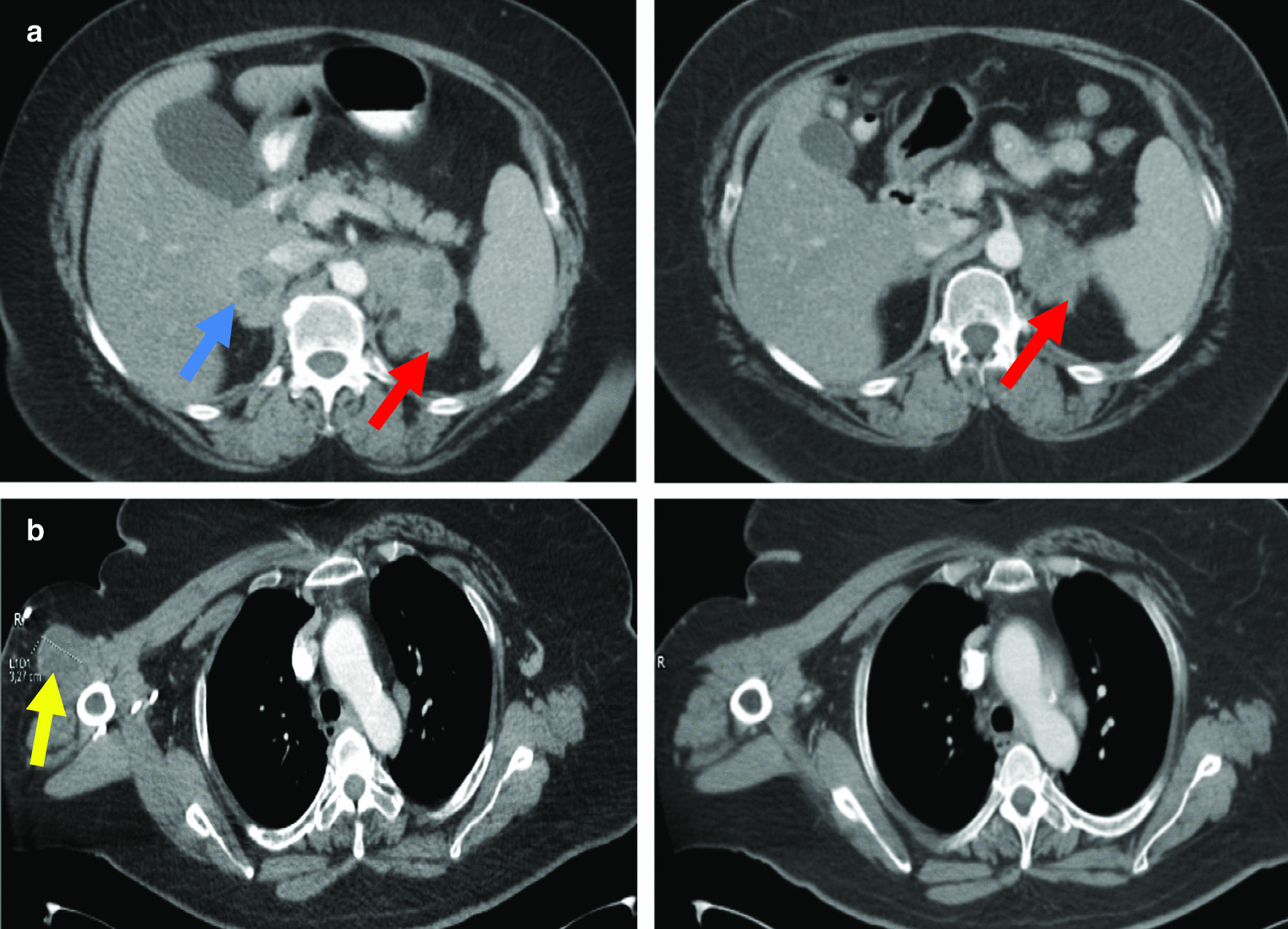
Disease response.** a** Partial response by Response Evaluation Criteria in Solid Tumors (in cancer immunotherapy trials) after 7 cycles of nivolumab, with disappearance of right adrenal metastasis and reduction of left suprarenal mass.** b** New metastasis at the right deltoid muscle (confirmed progressive disease by Response Evaluation Criteria in Solid Tumors, in cancer immunotherapy trials), vanishing on cisplatin plus pemetrexed rechallenge. Blue arrow points to right adrenal metastasis. Red arrow points to left adrenal metastasis. Yellow arrow points to right deltoid muscle metastasis

PD-L1 positivity, with the inference of a hypermutator phenotype, was considered to support the choice of immunotherapy with the patient’s agreement. Nivolumab 3 mg/kg intravenously every 2 weeks was administered for 38 cycles. After seven cycles, a PR by RECIST in cancer immunotherapy trials (iRECIST) was achieved (iPR). Immunotherapy was maintained for 19 months, from December 2016 to September 2018, with further confirmed PD iRECIST (iCPD) by solid biopsy of a new metastasis at the right deltoid muscle (Fig. 1).

The sample of this metastasis went into a multinational, prospective molecular screening program called ARCHE (OncoDNA S.A., Belgium), performing OncoDEEP™ comprehensive panel with 76 genes and personalized tumor immunogram. In the metastasis at the right deltoid muscle, the platform revealed PD-L1 positivity of greater than 50% with CD8^+^ T cells expression by IHC, and showed MSI with a deleterious c.298C>T (p.R100*) mutL homolog 1 (*MLH1*) gene mutation (variant allele frequency [VAF] 30%) (Table 2).

**Table 2:**
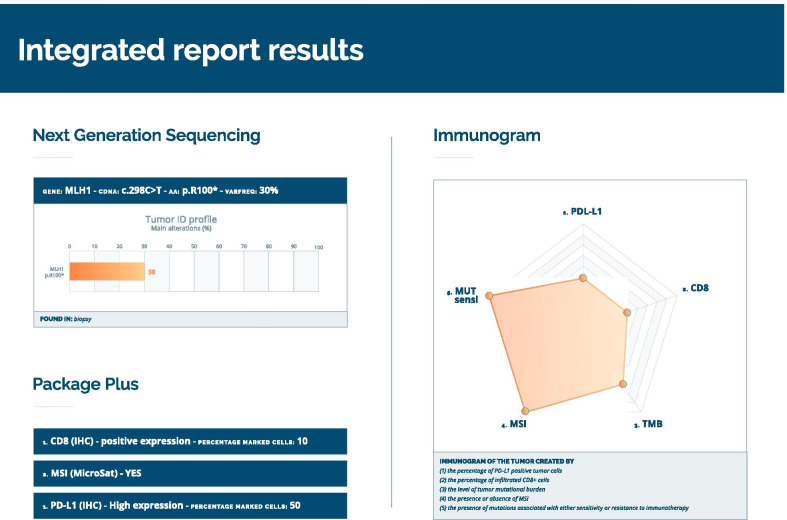
OncoDEEP™ Integrated report results

NGS comprehensive panel and personalized tumor immunogram results

In December 2018, a PR was achieved with cisplatin plus pemetrexed reintroduction since October 2018 (Fig. 1), but the disease progressed shortly after. Immunotherapy rechallenge and vinorelbine did not succeed either.

## Discussion

Mutations in *ARID1A* gene occur in a variety of tumors: gastric, endometrial, and clear cell ovarian cancers. Neoplasms formed by *ARID1A* deficiency have increased mutation rates, elevated tumor-infiltrating lymphocytes, and PD-L1 expression [2]. An 8% prevalence of *ARID1A* gene mutations has been described with next-generation sequencing (NGS) in lung adenocarcinoma samples [3]. In a Spanish cohort of 185 treatment-naïve patients, 12% of advanced lung adenocarcinoma cell-free DeoxyriboNucleic Acid (cfDNA) samples analyzed with Guardant360 harbored *ARID1A* mutations (61% pathogenic/likely pathogenic) [4]. To date, no clear epidemiological, clinicopathological, or molecular features have been specifically related to lung adenocarcinoma with *ARID1A* mutations.

In our patient, the PD-L1 positivity of 30%, and the presence of 97 genomic alterations (with five different exon deletions and one mutation in *ARID1A* gene accounting for 33.6% of altered cfDNA) (Table 1), made us estimate *ARID1A* aberrations as driver events.

The majority of *ARID1A* mutations are inactivating with loss of *ARID1A* expression, making them not easily druggable. However, molecular consequences of *ARID1A* deficiency in cancer may be exploited therapeutically. *ARID1A* interacts with MMR MutS protein homolog 2 (MSH2), recruiting MSH2 to chromatin during DeoxyriboNucleic Acid (DNA) replication, and promoting MMR. By contrast, *ARID1A* inactivation impairs MMR machinery leading to an increased mutation burden that correlates with MSI [2].

In deficient MMR cancers across 12 different histologies, overall response rate (ORR) of 53% (48/86) (95% CI 42–64) and complete response (CR) in 21% of patients, with a 2-year overall survival (OS) rate of 64%, were observed with anti-PD-1 antibody pembrolizumab [5]. Anti-PD-1 inhibitor nivolumab has shown an ORR of 36% (7% CR) in 42 patients with pretreated deficient MMR tumors [6]. However, no patients with non-small cell lung cancer (NSCLC) were included in these studies.

*ARID1A* mutations have been correlated with higher TMB (median 17.6 versus 7.4 mutations/Mb,* p* < 0.001) in microsatellite stable (MSS) tumors [7]. Conversely, in our tumor with MSI, the mutator phenotype could have been triggered by *ARID1A* driver alterations impairing MSH2 recruitment for DNA repair, without initial detection/no presence of MMR gene mutations, and with immunotherapy response due to MMR deficiency.

However, *ARID1A* has also been described as a causative gene for MSI through epigenetic silencing of the *MLH1* gene. Loss of *ARID1A* expression has been associated with sporadic MSI in endometrial carcinoma secondary to *MLH1* gene promoter methylation [8]. Since Guardant360 is not equipped for analyzing epigenomic changes, we hypothesized that *ARID1A* driver alterations could have also prompted this sporadic epigenomic pathway of MSI in our patient, leading to a high mutational rate.

**Fig. 2:**
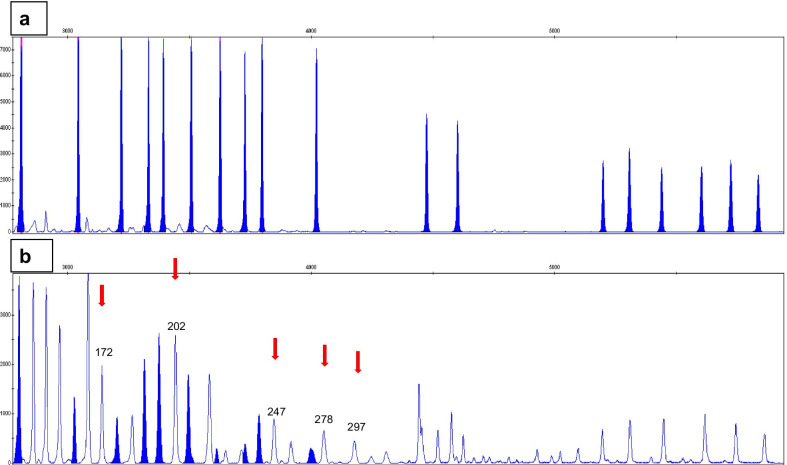
Genmapper of mutL homolog 1 (*MLH1*) methylation study. **a** Upper graph: Blood DNA pattern. **b** Bottom graph: Tumor DNA pattern, with peaks (red arrows and pair bases) marking the 5 methylation regions of *MLH1* gene

The OncoDEEP comprehensive panel in the deltoid tumor sample confirmed the suspected MSI, with a pathogenic *MLH1* gene mutation. In sporadic colon tumors with MSI, there is a high, but not complete, correlation between *MLH1* methylation and* BRAF* V600E mutation [9]. Likewise, the absence of* BRAF* V600E mutation could not definitively preclude an *MLH1* methylation in this lung adenocarcinoma. We proceeded to study *MLH1* gene promoter methylation (SALSA MS-MLPA Probemix ME011-C1 Mismatch Repair Genes) and its presence was unveiled in the deltoid metastasis (Fig. 2).

*MLH1* gene promoter methylation can follow novel patterns of inheritance [10]. Its germline prevalence in colorectal cancers (CRC) with MSI and loss of *MLH1* expression is 0.6% [11]. In our patient, we discarded the presence of the *MLH1* methylation at the germline setting. *MLH1* gene promoter methylation has also been described as a second allele inactivating hit in patients with *MLH1* germline mutations [9]. The VAF of the c.298C>T (p.R100*) *MLH1* gene mutation was not close to 50% for the inference of an inherited mutation [12], but tumor purity could have influenced VAF. We also ruled out a germline origin for the *MLH1* mutation, utterly excluding a Lynch syndrome diagnosis.

The VAF and its absence in the liquid biopsy do not confirm the *MLH1* gene variant as the first allelic event for MMR deficiency. Although not impossible, it is difficult to conceive a mutation with VAF of 30% in solid biopsy not detected in cfDNA. We consider that *MLH1* methylation was the first somatic hit and the *MLH1* gene mutation a second variant with, most probably, a low VAF when the liquid biopsy was taken which could have hindered its detection. The clonal evolution of a growing disease with MMR deficiency would have been accompanied by a rise in VAF of the *MLH1* gene mutation, mainly at the site of progression, enabling its identification in solid sample with OncoDEEP platform many months later.

To determine a possible immune response pattern in the resistant tumor biopsy, we reviewed the RNAseq data from the deltoid muscle metastasis. A high expression of C-X-C motif chemokine ligand 9 (*CXCL9*) gene was discovered, indicating the activation of the interferon-gamma pathway. Despite the persistence of this positive predictive biomarker along with MSI, and the lack of aberrations in resistance genes (Janus kinase 2 [*JAK2*], phosphatase and tensin homolog [*PTEN*], serine/threonine kinase 11 [*STK11*]), the patient ended up with tumor progression on immunotherapy.

In the current clinical scenario of progression, prioritization of therapy options for a tumor with MSI and other actionable genomic alterations in the original liquid biopsy is a challenging issue [13]. Categorizing molecular alterations according to therapeutic targets requires consideration of dominant signaling/repair pathways and scientific evidence.

In CRC with *MLH1* methylation and* BRAF* wild type, a 42% prevalence of actionable fusions has been reported [14]. Respectively 38% and 45.5% of MSI CRC harbor secondary* BRCA2* and* EGFR* mutations [15]. In our case, kinesin family member 5B-rearranged during transfection (*KIF5B*-*RET*) fusion, with higher VAF, could have been targeted over* BRCA* mutations (which need biallelic inactivation for actionability) and* EGFR* mutations. Tracking the evolution of targetable genomic alterations may help select the proliferating clone to be treated. Addressing the main stems of the disease is also appealing: *ARID1A* loss sensitizes cancer cells to poly(ADP-ribose) polymerase (PARP) inhibitors [16], and some agents have shown demethylating activity [17], providing candidate therapeutic opportunities for clinical trials.

To our knowledge, this is a unique case of lung adenocarcinoma with *ARID1A* gene alterations leading to sporadic MSI, through somatic *MLH1* epigenomic changes, with an *MLH1* gene mutation as the second somatic hit. Association of *ARID1A* mutations, MSI, high TMB, and PD-L1 expression contributes to more active immunotherapeutic responsiveness in advanced gastrointestinal cancers [18]. Coexistence of these molecular changes can also define a subset of lung adenocarcinoma with different prognosis and high vulnerability to immunotherapy. Although biomarker validation studies are encouraged, we suggest that *ARID1A* gene should be included in NGS panels used in metastatic lung adenocarcinoma. When *ARID1A* alterations are uncovered, MSI status should be known, even with comprehensive NGS platforms as well, to possibly predict durable antineoplastic response with immunotherapy.

Strikingly, with traditional hallmarks still leading to treatment response, we faced an usual case of PD after long-term immunotherapy. In the setting of MSI neoplasms, more research with complex tests based on solid and/or liquid biopsy are needed to find out primary and secondary resistance mechanisms to immune checkpoint inhibitors. After progression to immunotherapy in tumors with MSI, choosing the correct target among multiple actionable genomic aberrations is a difficult task. Development of therapies against *ARID1A* mutations and epigenomic methylation (when *MLH1* methylation is involved) is warranted.

## Conclusions

Reliable predictive biomarkers for sustained efficacy with targeted drugs have successfully emerged in recent years for metastatic lung adenocarcinoma. But in this era of precision oncology, more tumor molecular hallmarks related to systemic immunotherapy activity are urgently needed. The response to immune checkpoint inhibitors presented in this case report reflects that *ARID1A* genomic aberrations may contribute to this purpose, due to its relation to sporadic MSI through somatic* MLH1* epigenomic methylation. It is our opinion that *ARID1A* gene should be included in comprehensive NGS molecular platforms used in advanced lung adenocarcinoma. On the other hand, resistance to immunotherapy and lack of clearly validated biomarkers constitute a huge field for future research.

## Data Availability

All data analyzed during this study are included in this published article. The sequencing data generated and analyzed during the current study is available from the authors upon reasonable request and with permission of Guardant Health and OncoDNA.

## Abbreviations

*ARID1A*: AT-rich interactive domain-containing 1A
cfDNA: Cell-free DNA
CRC: Colorectal cancers
DNA: DeoxyriboNucleic Acid
FISH: Fluorescence in situ hybridization
iCPD: Confirmed progressive disease by iRECIST
IHC: Immunohistochemistry
iPR: Partial response by iRECIST
iRECIST: Response Evaluation Criteria in Solid Tumors, in cancer immunotherapy trials
*MLH1*: MutL homolog 1
MMR: Mismatch repair
MSI: Microsatellite instability
MSH2: MutS protein homolog 2
MSS: Microsatellite stable
NSCLC: Non-small cell lung cancer
NGS: Next-generation sequencing
ORR: Overall response rate
PD: Progressive disease
PD-L1: Programmed death ligand 1
PR: Partial response
RT-PCR: Real-time polymerase chain reaction
TMB: Tumor mutational burden
VAF: Variant allele frequency
